# Associated Factors and Mortality of Arrhythmia in Emergency Department: A Narrative Review

**DOI:** 10.7759/cureus.68645

**Published:** 2024-09-04

**Authors:** Utkarsh Gaur, Charuta Gadkari, Aditya Pundkar

**Affiliations:** 1 Emergency Medicine, Jawaharlal Nehru Medical College, Datta Meghe Institute of Higher Education and Research, Wardha, IND; 2 Orthopedics, Jawaharlal Nehru Medical College, Datta Meghe Institute of Higher Education and Research, Wardha, IND

**Keywords:** management, hypertension, risk factors, cardiovascular diseases, emergency department, mortality, arrhythmia

## Abstract

Cardiac arrhythmias represent a major concern in the emergency department (ED), particularly given their association with significant morbidity and mortality. This narrative review examines the various factors influencing arrhythmias and their impact on patient outcomes in emergency settings. Managing complex supraventricular and ventricular arrhythmias (VAs) during acute myocardial infarction (AMI) and severe cardiovascular conditions remains challenging, despite advancements in diagnostic and therapeutic techniques. Ventricular arrhythmias frequently forecast worse outcomes during hospital stays and heighten the chances of sudden cardiac death and cardiac arrest, especially within the initial 30 days after a heart attack. The incidence of arrhythmias in ED is increasing due to demographic changes and higher rates of chronic illnesses such as diabetes, hypertension, and chronic kidney disease. These comorbidities, coupled with lifestyle factors such as smoking and alcohol consumption, complicate arrhythmia management, especially among older adults and males. Rapid and precise ECG interpretation in the ED is crucial for identifying specific arrhythmia types and initiating appropriate treatments.

Atrial fibrillation (AF), the most prevalent form of rapid heart rhythm originating above the ventricles, notably impacts patient outcomes, particularly in cases of AMI and heart failure. In the ED, managing AF focuses on preventing strokes with thromboprophylaxis and employing risk assessment tools such as CHA2DS2-VASc and HAS-BLED scores. The analysis highlights how risk factors like hypertension, obesity, obstructive sleep apnea (OSA), and diabetes intricately influence the development and worsening of AF.

Optimizing AF treatment outcomes requires a multidisciplinary approach involving cardiologists, emergency physicians, and critical care specialists. Future research should prioritize evaluating the effectiveness of preventive and therapeutic interventions for AF, integrating new risk factors and genetic insights to enhance prediction and management strategies. Understanding the factors contributing to arrhythmias and mortality in the ED underscores the importance of timely and accurate diagnostic and therapeutic measures to improve patient care and outcomes.

## Introduction and background

Cardiovascular diseases remain a leading cause of natural death in developed nations despite advances in diagnostic technologies. Cardiac arrhythmias, which arise from abnormal electrical impulses in the heart, can result in irregular heartbeats, including bradycardic (slow) and tachycardic (fast) rhythms. Research from the Cleveland Clinic reveals that arrhythmias are responsible for over 50% of sudden cardiac arrests without preceding symptoms such as palpitations or chest pain [1]. The introduction of the Apple Watch for arrhythmia detection has identified arrhythmias in 2,161 out of 419,297 participants, though issues such as data accuracy, sensor reliability, and false positives persist [2].

Management of complex arrhythmias in emergency settings continues to be a significant challenge despite therapeutic advancements. Ventricular arrhythmias (VAs) are often linked to poorer in-hospital outcomes, yet the specific relationships between VA types, timing, treatment strategies, and prognosis in patients with acute myocardial infarction (AMI) remain poorly defined. This complexity also extends to the management of out-of-hospital cardiac arrest (OHCA) cases and the ongoing debate over the necessity of aggressive revascularization [3]. Although the frequency of VAs in patients with acute coronary syndrome (ACS) has decreased during hospitalization, the risk of cardiac arrest and sudden cardiac death (SCD) remains high, particularly within the first 30 days of post-myocardial infarction (MI) [4].

The rising prevalence of arrhythmias in emergency departments (EDs) reflects demographic shifts and an increase in chronic health conditions. Conditions such as coronary artery disease, heart failure, and previous MI predispose individuals to arrhythmias, while comorbidities like diabetes, hypertension, and chronic kidney disease further complicate management [3,5]. Demographic factors, including age and sex, also play a critical role, with older adults and males being particularly vulnerable due to age-related cardiac changes and lifestyle factors like smoking and alcohol consumption [5].

In the ED, rapid and accurate ECG interpretation is crucial for diagnosing arrhythmias and guiding treatment. Bradycardic and tachycardic rhythms, in particular, pose high mortality risks due to their potential to cause sudden cardiac arrest [3]. Management strategies in the ED include pharmacological interventions, electrical cardioversion, and, when necessary, catheter ablation. The effectiveness of these treatments depends on timely administration and the patient's overall condition. A multidisciplinary approach involving cardiologists, emergency physicians, and critical care specialists is essential to optimize treatment outcomes [5]. This review seeks to synthesize current knowledge on the factors associated with arrhythmias and assess their impact on mortality rates among patients presenting to the ED.

## Review

Methodology

This review article systematically assessed published studies to provide a comprehensive discussion of the factors influencing arrhythmias and associated mortality, primarily focusing on ED settings. However, studies involving non-ED patients were also considered if they provided relevant insights. A rigorous search was conducted across multiple databases, including PubMed, Web of Science, and Google Scholar, using key terms such as 'arrhythmia,' 'mortality,' 'emergency department,' 'cardiovascular diseases,' 'risk factors,' 'hypertension,' and 'management.' Studies were included if they discussed factors leading to arrhythmias, while those with poor methodological quality, outdated data, or insufficient information were excluded. From this process, 52 relevant papers published between 1983 and 2021 were selected for inclusion in this review.

Atrial fibrillation and its risk factors

Atrial fibrillation (AF), the most common arrhythmia, can present without symptoms or cause sudden hemodynamic instability requiring urgent medical care. Approximately 20% of patients with AMI have a history of AF, and 5% of those experiencing ST-elevation MI develop new-onset AF [6]. Among AMI patients undergoing primary percutaneous coronary intervention, those who develop AF face increased mortality and complications compared to those without AF. Atrial fibrillation affects approximately 1% to 2% of the general population and 9% of individuals over 80 years old [6]. In the ED, most cases of AF are stable from a hemodynamic standpoint. However, rapid conduction of AF can lead to hemodynamic instability. It can also occur acutely in emergencies such as acute coronary syndrome (ACS), acute heart failure exacerbation, sepsis, and other critical conditions. In emergency settings, stroke prevention should be prioritized based on the patient's condition. Thromboprophylaxis, whether oral or intravenous, should be considered for AF cases, with risk stratification guided by CHA2DS2-VASc and HAS-BLED scores [7]. Recently, a comprehensive systematic review in an Agency for Healthcare Research and Quality (AHRQ) review (2013) concluded that CHA2DS2-VASc and HAS-BLED scores for risk stratification are useful [8].

Prevalence and prognosis in AMI patients

Heart failure and AF commonly coexist, worsening the prognosis of both conditions. Among ST-elevation MI patients, approximately 9% develop AF during or shortly after percutaneous coronary intervention. Other prevalent supraventricular arrhythmias that occur after angioplasty in MI patients include sinus bradycardia (28%) and sinus tachycardia (22%) [9]. In a community-based study involving 3,220 patients with incident MI, 9% had AF prior to their MI [10]. During follow-up, AF developed in 23% of patients, with 7% experiencing onset within two days and an additional 4% within 30 days post-MI. The onset of AF was linked to a notable increase in mortality, except for those who developed AF within the first two days of their MI.

Pre-excitation syndromes

Pre-excitation occurs when an accessory pathway conducts in the antegrade direction, potentially causing arrhythmias. The most common arrhythmia resulting from this is orthodromic atrioventricular re-entrant tachycardia (AVRT). Additionally, an accessory pathway may lead to fibrillation, flutter, or tachyarrhythmias of the atrium. The absence of decremental conduction properties in the accessory pathway results in rapid conduction, which may induce very swift ventricular responses, potentially causing hemodynamic compromise or progressing to ventricular fibrillation (VF), thus elevating the risk of SCD [11]. Occasionally, this could represent the initial presentation of pre-excitation syndromes.

All arrhythmias associated with accessory pathways have the potential to become medical emergencies. When these arrhythmias lead to hemodynamic compromise, immediate intervention is critical, often requiring electric cardioversion [12]. For stable patients, medical management may involve using agents to block or suppress accessory pathway conduction and/or facilitate the restoration of sinus rhythm.

Cardiovascular risk factors for AF

Atrial fibrillation is influenced by several cardiovascular risk factors, including aging, diabetes, hypertension, congestive heart failure, coronary artery disease, and valvular heart disease [13,14]. Additional risks include male sex, obesity, excessive alcohol intake, and left ventricular hypertrophy [15]. Preventive strategies focus on addressing these factors early. For example, significant weight loss following bariatric surgery can reduce AF risk by 20% in obese individuals, though lifestyle changes show mixed results [16]. Controlling hypertension, high BMI, diabetes, and smoking could prevent more than half of AF cases in middle-aged adults, with AF prevalence rising with the number of metabolic syndrome components [17].

Emerging risk factors and research

Recent research has identified new risk factors for AF that are not yet fully integrated into mainstream studies. Increased pulse pressure, often resulting from aortic stiffness, is linked to a higher risk of AF [18]. Obstructive sleep apnea (OSA) is prevalent among AF patients and independently increases AF risk beyond what is attributable to obesity. Furthermore, a sedentary lifestyle, poor cardiorespiratory fitness, and excessive high-intensity endurance training contribute to rising AF rates [19,20]. Genetic studies have also uncovered both common and rare gene variations contributing to AF's hereditary aspect. This has led to a reduction in cases previously labeled as 'lone atrial fibrillation' to less than 2%, challenging the term's use in clinical contexts [21].

Obesity

Obesity in sheep leads to various structural and electrophysiological changes, including left ventricular hypertrophy, diastolic dysfunction, increased left atrial volume, fibrosis, elevated fat content, reduced left atrial conduction velocity, and prolonged AF duration [22]. Prolonged obesity (72 weeks) in sheep creates a specific AF condition marked by epicardial fat infiltration into the posterior left atrium, which lowers endocardial voltage [23,24]. Recent studies, particularly in rats, indicate that a high-fat diet can extend AF duration by slowing atrial conduction and reducing pulmonary vein refractoriness, regardless of obesity [25,26].

Further investigations into adipose tissue's cellular effects on atrial cardiomyocytes reveal that exposure to adipocytes in rabbits increases action potential duration, induces spontaneous activity with after-depolarizations under adrenergic stimulation, and alters several ion currents [27,28]. These findings suggest that both obesity and high-fat diets contribute to atrial electrophysiological changes, which can promote the development and persistence of AF, highlighting the need for further research into the mechanisms underlying these effects.

Obstructive Sleep Apnea

Acute OSA, induced with endotracheal tubes, increases venous pressure, activates ganglionated plexi, and results in left atrial dilation, shortened atrial refractory periods, and abnormal intraventricular flow [29-31]. Treatments such as ganglionated plexi ablation, atropine, or vagotomy can modulate these effects. Additionally, OSA-induced hypercapnia prolongs atrial refractory periods and slows conduction, persistently increasing abnormal flow velocities [29,30].

Chronic OSA in humans over weeks also leads to structural changes such as atrial fibrosis and altered connexin 43 distribution, contributing to atrial electrical abnormalities and a predisposition to atrial fibrillation [31,32]. These findings highlight the significant impact of both acute and chronic OSA on atrial structure and function, emphasizing the need for effective treatment strategies to mitigate these changes and reduce the risk of AF.

Hypertension

Experimental models have shown that hypertension-related abnormal atrial substrates include left atrial enlargement, atrial fibrosis, increased AF vulnerability and duration, delayed conduction, increased conduction heterogeneity, reduced expression of connexin 43, and variable changes in atrial effective refractory periods (ERP) [33-35]. These structural and electrophysiological alterations contribute to the heightened risk of AF in hypertensive conditions.

Additionally, a study on rats highlighted significant changes in calcium handling. These changes included decreased expression of CACNA1C, reduced levels of ryanodine receptor type 2 (RyR2), and lower Na+/Ca2+ exchanger expression [36]. These alterations were accompanied by an increased calcium load in the cells and intracellular calcium (Ca2+) alternans, further contributing to the arrhythmogenic potential in hypertensive atrial substrates [36].

Diabetes Mellitus

Research on atrial remodeling in diabetic models is relatively sparse compared to studies on other related factors. In rats with streptozotocin-induced diabetes mellitus, findings include reduced phosphorylation of atrial connexin 43 and increased fibrosis [37]. There is also a notable rise in receptors for advanced glycation end products (AGEs) and AGEs themselves. Interestingly, inhibiting the production of AGEs has been shown to prevent the development of fibrosis, highlighting a potential therapeutic target for mitigating atrial remodeling in diabetes [38].

Additionally, rabbits with alloxan-induced diabetes mellitus have demonstrated significant atrial fibrosis, an increased risk of AF, and slowed atrial conduction [39]. These findings underscore the impact of diabetes on atrial structural and electrical properties, contributing to a higher susceptibility to AF. The studies suggest that diabetes-induced changes in the atria, such as fibrosis and altered conduction, play a crucial role in the development of AF, emphasizing the need for further research in this area to explore potential interventions and therapeutic strategies [39].

Impact of lifestyle factors on atrial remodeling

Recent studies have examined the influence of lifestyle-related risk factors on atrial remodeling. Acute alcohol consumption in healthy canine hearts did not significantly affect atrial effective refractory period (ERP), conduction, or susceptibility to AF [40]. However, in rabbit atria, a 120-hour ethanol infusion led to a decrease in L-type calcium current density [41]. Cellular models from pulmonary veins demonstrated that alcohol shortened action potential duration without increasing delayed after-depolarizations. The long-term effects of chronic alcohol consumption on atrial structure remain to be fully understood and warrant further investigation. In contrast, a study using a rat model indicated that long-term endurance training resulted in significant changes, such as atrial fibrosis, dilation, and autonomic alterations, which increased atrial vulnerability [42].

Risk factors influencing the recurrence of AF

The development of abnormal atrial structures and the progression of AF are closely linked to various risk factors. Aging, left atrial enlargement, and congestive heart failure are commonly associated with AF progression [43]. Research has shown that a history of cerebrovascular disease, chronic obstructive pulmonary disease (COPD), hypertension, and other conditions also predict AF progression [44,45]. These factors contribute to the declining efficacy of catheter ablation over time, as studies indicate that abnormal atrial substrates continue to develop despite previous successful ablations. This ongoing remodeling complicates rhythm control and highlights the need for a comprehensive management approach that addresses these underlying risk factors [44]. Understanding these predictors can aid in developing more effective long-term treatment strategies and improving patient outcomes [45].

Studies on rhythm control strategies have identified several independent predictors of AF recurrence. Jacobs et al. [46] found that among 2,179 patients undergoing catheter ablation, higher CHADS2 and CHA2DS2-VASc scores were associated with an increased risk of AF recurrence five years after the procedure. Additionally, patients with metabolic syndrome components, even without AF, showed reduced survival rates post-ablation. Obesity is also linked to AF recurrence, with a meta-analysis indicating that every 5-unit increase in BMI correlates with a 10% higher risk in postoperative settings and a 13% higher risk in post-ablation settings. Furthermore, pericardial or epicardial fat may serve as a specific predictor for AF recurrence risk after catheter ablation [47].

Obstructive sleep apnea significantly increases the risk of AF recurrence following pulmonary vein isolation, with a meta-analysis of nearly 4,000 patients showing a 25% higher recurrence rate [48]. This underscores the importance of managing OSA to improve long-term outcomes. While the evidence on hypertension as a predictor of AF recurrence is mixed, long-term studies consistently identify hypertension as an independent prognostic factor, emphasizing the need for effective blood pressure management to reduce AF recurrence risk [49].

Aortic stiffness, characterized by reduced aortic elasticity, has also been associated with poorer rhythm control outcomes after catheter ablation in patients with lone AF [50]. Although evidence on diabetes mellitus as a predictor of arrhythmia recurrence is limited, recent studies found that higher HbA1c levels post-ablation correlated with an increased risk of AF recurrence, suggesting that better glycemic control might help prevent recurrence [51]. Moreover, alcohol consumption has been linked to atrial remodeling, leading to higher AF recurrence rates in patients who have undergone catheter ablation [52]. These findings highlight the multifaceted nature of AF recurrence and the need for a comprehensive approach that includes managing OSA, hypertension, aortic stiffness, glycemic control, and lifestyle factors to enhance the effectiveness of AF treatments.

## Conclusions

Managing arrhythmias in emergency departments is complex, influenced by patient demographics, underlying health conditions, and the nature of the arrhythmias. A detailed review underscores these factors' impact on mortality rates, stressing the importance of prompt and accurate diagnosis and treatment interventions. Cardiac arrhythmias remain a serious threat, especially during AMI and other severe cardiovascular conditions. Ventricular arrhythmias during AMI are particularly concerning due to their association with worse outcomes and higher mortality rates in hospitals. Despite advances in treatment, managing these arrhythmias in emergencies remains challenging. The first 30 days post-AMI are critical, with increased risks of sudden cardiac death and cardiac arrest, highlighting the importance of vigilant monitoring and prompt intervention.

Arrhythmias in emergency departments are worsened by demographic changes and increasing chronic illnesses. Conditions such as diabetes, hypertension, and chronic kidney disease heighten both the occurrence and complexity of arrhythmias. Lifestyle factors such as smoking and alcohol use amplify these risks, particularly in older adults and males vulnerable to age-related heart changes. Swift and precise ECG analysis is crucial in the emergency setting for timely diagnosis and treatment initiation.

Atrial fibrillation, the most common arrhythmia, significantly affects patient outcomes, especially in AMI and heart failure cases. Recognizing how risk factors such as hypertension, obesity, OSA, and diabetes interplay is vital for understanding AF development and progression. This understanding guides effective prevention and treatment strategies. Future research should focus on assessing interventions to prevent AF, especially in high-risk populations. Incorporating new risk factors and genetic insights into clinical practice could enhance arrhythmia prediction and management, improving patient outcomes in emergency departments.
